# Efficacy of Chlorhexidine Impregnated Wipes for the Local Dysbiosis in Atopic Dogs: A Multicentric Prospective Study

**DOI:** 10.3390/vetsci11060240

**Published:** 2024-05-27

**Authors:** Emmanuel Bensignor, Christelle Navarro, Carole Gard, Bruno Jahier, Charline Pressanti, Emilie Videmont

**Affiliations:** 1 Dermatoveto, 17 Boulevard des Filles du Calvaire, F-75003 Paris, France; emmanuel.bensignor@wanadoo.fr; 2 MP Labo, 45 Boulevard Marcel Pagnol, F-06130 Grasse, France; 3 Dermatology Unit, Departement of Clinical Sciences, Université de Toulouse, ENVT, F-31300 Toulouse, France; 4 Dermatology Unit, CHV Saint Martin, F-74350 Allonzier La Caille, France

**Keywords:** atopic dermatitis, wipes, skin dysbiosis, chlorhexidine, antiseptics

## Abstract

**Simple Summary:**

Canine atopic dermatitis (CAD) is a frequent skin disease in dogs in which a microbial dysbiosis contributes to flare ups. The use of topical products for localised areas of dermatitis is useful as it can reduce the use of antibiotics and the development of antimicrobial resistance. This study aimed to evaluate the benefits of chlorhexidine wipes in case of localised dysbiosis in atopic dogs. Twenty dogs received a daily application of wipes for 14 days and showed a clinical and cytological statistically significant improvement following the use of the tested product after 7 and 14 days of application. Antiseptic wipes are beneficial in case of localised dysbiosis in CAD.

**Abstract:**

(1) Background: Dysbiosis is frequently observed in Canine Atopic Dermatitis (CAD). Antimicrobial treatment may be necessary to treat flare ups and the use of topical treatments is beneficial to prevent the development of bacterial resistance. Wipes are an easy way to apply antiseptic agents on the skin. The aim of this study was to evaluate the benefits of 3% chlorhexidine impregnated wipes (Pyoskin^®^ wipes, MP Labo, France) on local areas of dysbiosis in dogs with CAD. (2) Methods: A total of 20 dogs suffering from CAD presented with localised areas of dysbiosis were included in this study. Affected areas were cleansed with the daily application of chlorhexidine wipes once a day for 14 days. Follow-up visits were scheduled after one and two weeks. Clinical signs (lesions and pruritus), dysbiosis scored by cytological counts (cocci and *Malassezia*) and investigator and owner global appreciation were evaluated. (3) Results: A statistically significant decrease in clinical scores and cytological counts were observed as soon as D7 and until D14. Both owner and investigator appreciation were considered high (4) Conclusions: The use of chlorhexidine impregnated wipes is a useful and easy way to manage localised dysbiosis in atopic dogs and allows limiting of systemic medication to prevent bacterial resistance.

## 1. Introduction

Canine atopic dermatitis (CAD) is very frequently associated with secondary microbial infections, such as bacterial overgrowth, pyoderma and *Malassezia* dermatitis [1]. These microbial complications are linked to local disturbances of the microbiota and defective receptors in atopic skin and defective production of antimicrobial peptides by keratinocytes [2]. This increase in numbers of *Staphylococcus* spp. and/or *Malassezia* spp. is a hallmark of atopic skin in dogs as in humans [3]. If not managed appropriately, this dysbiosis can then lead to the development of bacterial and yeasts infections, which play an important role in the worsening of clinical signs or inducing flare ups, as bacteria and *Malassezia* increase the production of pruritogenic and inflammatory cytokines which are responsible for a vicious cycle aggravating pruritus [4]. 

Recognising and managing bacterial and fungal dysbiosis is mandatory early in the management of CAD, not only to restore the skin microbiota but also to limit the development of infections, which may require systemic antimicrobials and increase the risk of the spread of antibiotic resistance [5]. 

Topical antiseptics are usually effective for the management of localised bacterial and/or *Malassezia* overgrowth [6,7] because they allow the direct delivery of active ingredients to the skin at high concentrations and are a useful addition to systemic medications [8,9]. The use of easy to deliver formulations is effective in improving compliance and increase the likelihood of success. Wipes are particularly suitable as they allow a mechanical removal of dirt and an impregnation of the affected areas with the ingredients [10]. This formulation is particularly convenient for difficult to reach areas such as skin folds and interdigital spaces and/or in case of compliance issues. 

Among the various antimicrobial agents available, chlorhexidine has strong evidence of efficacy for *Staphylococcus* spp. and to a lesser extent for *Malassezia* spp. [11]. Therefore, the aims of this study were to evaluate the efficacy and safety of wipes impregnated with 3% chlorhexidine on localised areas of microbial dysbiosis in dogs suffering from atopic dermatitis. 

## 2. Materials and Methods

This was a multicentric, prospective open label study carried out by three specialists, diplomates from the European College of Veterinary Dermatology, in accordance with the European convention for the protection of vertebrate animals used for experimental and other scientific purposes (n°123) and the European directive regarding the protection of animals used for scientific purposes (Directive 1986/609/CE) and approved by the ethical committee of Toulouse National Veterinary School under license SSA-2023-008. A signed owner consent was obtained for each dog included in the trial. 

Dogs were enrolled if they presented a non-seasonal atopic dermatitis associated with localised lesions of dysbiosis. Diagnosis of CAD was made on the basis of compatible history, presence of at least six clinical signs of the diagnostic criteria of Favrot [12], after exclusion of other pruritic skin disease (such as scabies, demodicosis and flea infestation by skin scrapings and combings), bacterial pyoderma by impression smears (absence of neutrophiles), flea allergy dermatitis by a rigorous flea control and food allergy by an adapted elimination diet during at least 8 weeks prior to enrolment. 

Dysbiosis was confirmed by a cytological examination using the stained tape strip technique allowing the microscopical detection of an overgrowth of bacteria and/or Malassezia, counted on 10 consecutive oil immersion fields at 1000× magnification. To be included, mean counts of more than 15 coccis per field [8] and/or 3 Malassezia per field [9] were needed. In case of multiple lesions, the most affected area was selected for sampling and counting microbial populations. 

To be included, dogs had to be clear of treatment with antibiotics or azole derivatives for 1 month, short-acting steroids for 2 weeks, long-acting steroids for 6 weeks, and any topical medication for one week prior to the start of the study. Dogs treated with oclacitinib, lokivetmab, ciclosporine, antihistamine, free fatty acids and/or hypo-sensitization were included only if the medication regimen was not modified 8 weeks before and during the study. 

Wipes containing 3% chlorhexidine, glycerine, allantoin and coco-glucoside (Pyoskin^®^ wipes, MP Labo, Grasse, France), were applied for 14 days once a day for 10 s on the affected skin to allow a correct penetration of the product. The dog was then left to dry naturally. To improve compliance and the effectiveness of the tested product, the first application was made at the clinic by the investigator. No other concomitant treatment was allowed during the course of the study except for the administration of an oral flea treatment with an isoxazolide molecule if needed to maintain a correct flea preventative program. 

Dogs were evaluated at D0 (inclusion), D7 (+/−1 day) and D14 (+/−1 day). At each visit a clinical and cytological score were calculated. Furthermore, at D14 an owner and veterinarian global assessment of efficacy (OGATE) and an owner satisfaction questionnaire were collected. 

The same area was assessed at each visit. Clinical scoring was based on a calculation of lesion severity (0 absence, 1 mild, 2 moderate, 3 severe and 4 very severe [13]) and extent (0 absent, 1 size less than a square of 4 cm, 2 size less than a square of 9 cm, 3 size less than a square of 16 cm, 4 size less than a square of 25 cm and 5 size more than a square of 25 cm, adapted from [10]). Pruritus was evaluated using a validated visual linear analogue scale ranging from 0 (no pruritus) to 10 (very severe pruritus) [14]. Dysbiosis scoring was performed at each visit. At the last visit, both the investigator and the owner were asked to globally appreciate the response to the wipes from 0 (no response) to 4 (excellent response) [15]. Finally, the owner was asked to fill a questionnaire to assess the practicability of the protocol and the cosmetic characteristics of the wipes (fragrance, skin hydration, level of moisture, level of cleaning efficacy, texture, size, and resistance) on a visual linear scale from 0 (very poor) to 10 (very good). 

Statistical evaluations were performed using Statgraphics^®^ Centurion (version XVI.II) software. A generalized linear mixed-effects model (fixed effect: time and random effect: animal) was used for the analysis of the evolution of the cocci, Malassezia and total microbial counts, clinical and pruritus scores over time. In case of significance, post hoc pairwise comparisons were performed using the Fisher’s least significant difference (LSD) procedure. Ninety five percent confidence interval and *p*-value were provided to analyse the significance of the least square means estimates. A Pearson’s correlation test was performed to assess the correlation between the investigator and owner global evaluation at D14. Primary outcomes were the mean number of bacteria and yeast improvement and percentage reduction of microbial counts at the different time points. Secondary outcomes were the improvement of clinical and pruritus scores at each time point compared to baseline value.

## 3. Results

### 3.1. Animals

The study included 21 dogs, one of which was lost to follow-up after D0 and was not evaluated in the calculations. Therefore 20 dogs completed the study: 13 different breeds (French Bulldog 4, Crossbred 3, Labrador retriever 2 and American Staffordshire terrier, Border terrier, Bull terrier, Cavalier King Charles Spaniel, Chihuahua, Dalmatian, English Bulldog, English Cocker spaniel, Rottweiler, Shiba Inu and Shi Tzu; one of each) of a mean age of 5.3 years (1–13 years), 14 females (8 spayed) and 6 males (3 spayed) with a mean bodyweight of 18.49 kg (+/−11.5 kg). Dogs were diagnosed with atopic dermatitis on average 924 days prior to the study (0–3204 days) and the average duration of the current episode was 21.7 days (7–60 days).

### 3.2. Lesions

The number of lesions varied between 1 only (5 cases) and up to 8 different areas (1 case). Lesions were localised on different zones; the most affected (studied) areas were the paws (11 cases, 8 front and 3 hind), the face (3 cases), the peri-vulvar area (2 cases) and the lip, the ventral neck, the stomach, and the ventral part of the tail (1 case each). 

Severity scores varied between 2.0 and 3.0 at D0 depending on the location of the lesions with an average of 2.35 (+/−0.49). After treatment, average scores improved: respectively 1.6 (+/−0.6) and 1.15 (+/−0.59) at D7 and D14 (*p* < 0.001). Extent of lesions varied between 1.0 and 4.0 at inclusion with an average score of 1.65 (+/−0.75), improving over time (respectively 1.45 +/− 0.6 and 1.2 +/− 0.6 at D7 and D14). A statistically significant difference was observed only between D14 and D0 (*p* < 0.01). 

**Figure 1:**
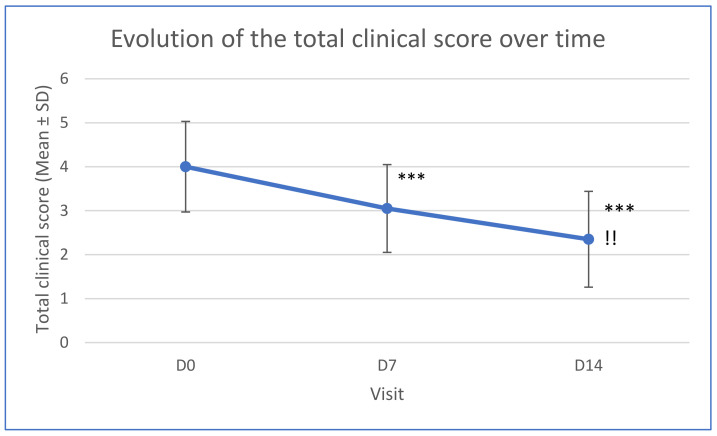
Evolution of the total clinical score over time (*** significant difference compared to D0 *p* < 0.001; !! significant difference compared to D7 *p* < 0.01).

Total clinical score also improved over time, with a significant difference observed between D7 and D0 (*p* < 0.001), D14 and D0 (*p* < 0.001) and D14 and D7 (*p* < 0.01) (Figure 1). 

Pruritus score varied at inclusion between 2.5 and 9.0 with an average of 5.18 +/− 1.86) and decreased to 3.63 +/− 1.71 at D7 (*p* < 0.01) and 2.75 +/− 2.57 at D14 versus D0 (*p* < 0.001). A significant improvement was also noted between D7 and D14 (*p* < 0.01).

### 3.3. Dysbiosis

**Figure 2:**
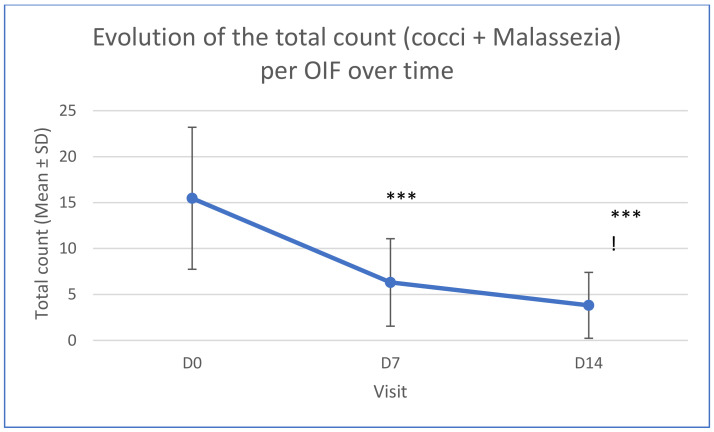
Evolution of the total microbial count over time (*** significant difference compared to D0 *p* < 0.001; ! significant difference compared to D7 *p* < 0.05); OIF: Oil Immersion Field.

Dysbiosis was observed for all cases. Average per field at inclusion was 10.51 +/− 9.05 for cocci and 4.96 +/− 4.38 for Malassezia. A decrease in microbial populations was observed both for cocci and yeasts after one week (*p* < 0.001) and further after two weeks (*p* < 0.001). The decrease was rapidly observed following treatment. Respectively 8 (40%) and 10 (50%) dogs presented a decrease of more than 75% of the cocci and the Malassezia counts at D14. Table 1 and Figure 2 show the evolution of microbial counts over time following treatment. 

### 3.4. Investigator and Owner’s Assessments

Mean scores of global evaluations were 3.1 (+/−0.8) and 2.6 (+/−0.9) for owners and investigators, respectively. In only one case did the investigator (but not the owner) consider the response to be weak. The efficacy was considered to be excellent for four and six cases for investigators and owners, respectively. 

Wipes were considered very easy to use (score 3.6/4), with good skin cleaning properties (score 8.7/10). Some owners considered the wipes not to be wet enough. 

**Figure 3:**
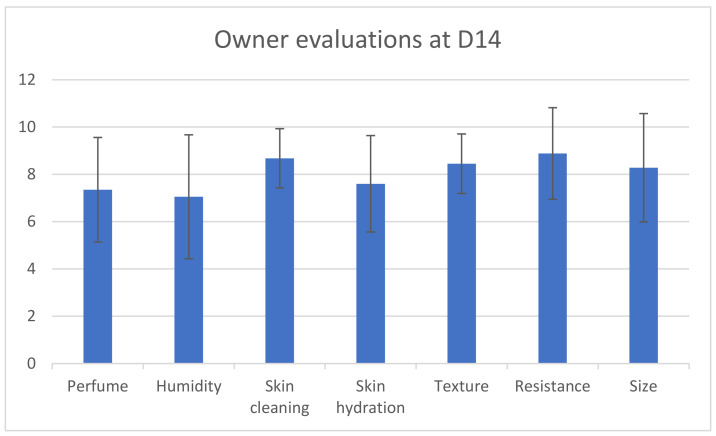
Owner’s evaluations at D14 (0-very bad to 10-excellent).

Additionally, the owner evaluations at D14 indicated a mean score of 7.4 (±2.2) for perfume, suggesting satisfactory acceptance of the odour by owners. Furthermore, the mean score for skin hydration at D14 was 7.6 (±2.0), indicating a moisturizing effect of the wipes, which may be beneficial for dogs with atopic dermatitis (Figure 3).

### 3.5. Safety

No adverse effects were reported during the course of the study. 

## 4. Discussion

The results of this open multicentric prospective field study confirm that the application of wipes impregnated with 3% chlorhexidine is an interesting approach to manage localised dysbiosis in CAD. 

Dysbiosis is a fundamental element in the pathogenesis of canine atopic dermatitis, as stated in the new disease definition proposed by the International Committee on Allergic diseases of Animals (ICADA): “canine atopic dermatitis is a hereditary, generally pruritic and predominantly T-cell-driven inflammatory skin disease involving interplay between skin barrier abnormalities, allergen sensitisation and microbial dysbiosis” [16]. Alterations of bacterial diversity and loss of cutaneous microbial diversity is associated with an increase in numbers of *Staphylococcus pseudintermedius* (and probably *Malassezia* sp.) correlated with the severity of the clinical signs of CAD and is strongly associated with the development of bacterial overgrowth, superficial and less commonly deep bacterial pyoderma, as well as *Malassezia* dermatitis in atopic dogs [17]. Bacteria and *Malassezia* stimulate flare ups of CAD by increasing the production of various inflammatory and pruritogenic cytokines and microbial allergens. Dysbiosis is also responsible for altered skin barrier in CAD (measured by trans-epidermal water loss, pH, and stratum corneum hydration) which can be responsible for an increase in severity of skin lesions [17].

Early management of local disturbances of the microbiota is therefore an important step in the management of CAD. Some studies have shown that systemic antibiotic, antifungal and/or antipruritic treatments are effective in improving clinical signs in atopic dogs and restore bacterial diversity in dogs with AD [17,18]. The use of topical antiseptics is particularly interesting as it can decrease the need for use of antimicrobial treatments, such as antibiotics or antifungal agents, and therefore the risk of developing antimicrobial resistance. It has been demonstrated that the use of topical formulations containing chlorhexidine is effective as a first line aid, as they deliver ingredients directly to the skin where pathogens live [11]. 

Previous in vitro data supported the effectiveness of the tested wipes both for bacteria and yeasts as they allowed a reduction of more than log5 in 1 min for standardized strains of bacteria and of more than log4 in 5 min for *Malassezia* from dilution to 10% [19]. Our in vivo data confirm this effectiveness as both total counts of coccis and *Malassezia* decreased rapidly after the use of the wipes as soon as after one week, and further after two weeks following application. This quick activity of chlorhexidine was observed in previous studies evaluating different formulations and vehicles [9,10,20,21]. Clinical signs (lesions and pruritus) also improved rapidly following wipe application, with a significantly statistical difference observed as soon as after D7 and further at D14. Improvement of pruritus may be linked with the antiseptic activity of chlorhexidine and/or with the soothing effect of the wipes, as previously suggested [10]. The primary limitation of this open prospective study lies in the absence of a control group. Therefore, these results should be confirmed by a double-blinded randomised study incorporating placebo wipes (without chlorhexidine).

The use of wipes for localised lesions is particularly interesting for a number of reasons. Firstly, they are easier to apply than shampoos; secondly, they do not require rinsing, which improves owner compliance; thirdly, they have a mechanical “rubbing” effect, which improves their effectiveness compared to lotions or sprays; and finally, they are easier to use in hard-to-reach areas, such as interdigital spaces or folds. As these areas are prone to lesions in CAD, wipes are ideal for atopic dogs. 

The wipes used in this study are fragrance-free, a deliberate choice aimed at minimising the introduction of potentially sensitising substances to already compromised skin [22]. Despite the absence of fragrance, the satisfaction rating of 7.4 out of 10 suggests that the lack of scent in the wipes did not lead to owner dissatisfaction. This observation underscores the importance of minimising unnecessary additives in products intended for use on dermatologically sensitive subjects. It highlights the efficacy of fragrance-free formulations in maintaining overall satisfaction without compromising the performance of the product. Moreover, the mean score at D14 for the ability of the wipes to moisturize the skin was 7.6 (±2.0), indicating a notable hydrating effect. This finding holds significant clinical relevance, particularly for dogs with atopic dermatitis, where maintaining skin hydration is paramount. Glycerine and allantoin, key components of the wipes, likely contribute to this moisturising effect, counterbalancing the potential drying effect of chlorhexidine. This is particularly beneficial for atopic dogs, whose compromised skin barrier and predisposition to dehydration demand careful consideration of moisturisation strategies. Studies have demonstrated the hydrating properties of both glycerine and allantoin, emphasising their importance in formulations targeting skin health and integrity [23,24]. Therefore, the inclusion of these ingredients in chlorhexidine wipes presents a synergistic approach to managing dysbiosis while supporting skin hydration, crucial in the management of atopic dermatitis. The wipes used in this study were well tolerated as no adverse effect was reported over 14 days. While antiseptics are typically favoured for addressing acute flares of atopic dermatitis, long-term management of chronic cases necessitates a comprehensive approach focused on enhancing skin and coat hygiene, to reinforce the skin barrier and limit the development of skin dysbiosis [25,26].

## 5. Conclusions

The use of chlorhexidine impregnated wipes is a useful and easy way to manage localised dysbiosis in atopic dogs and allows limiting of systemic medication to prevent bacterial resistance. The observed clinical and cytological improvements confirm the potential interest of this topical approach, with no reported adverse effects further supporting its safety profile. Reported satisfaction by owners and investigators, alongside the ease of use and positive cosmetic characteristics of the wipes, indicates reasonable acceptance among dog owners, despite the absence of fragrance. Additionally, the inclusion of moisturising components may offer benefits in supporting skin hydration, an essential consideration for skin integrity in atopic dogs. In summary, chlorhexidine-impregnated wipes offer a practical option for managing localised dysbiosis in atopic dogs, contributing to a comprehensive approach to the management of canine atopic dermatitis.

## Figures and Tables

**Table 1:** Evolution of total microbial counts over time.

Total Counts	Average	Median	SD
D0	15.47	15.5	7.73
D7	6.31	6.15	4.76
D14	3.82	2.35	3.59

## Data Availability

Data are available upon request to carole.gard@destaing.com.
